# False positive morphologic diagnoses at the anomaly scan: marginal or real problem, a population-based cohort study

**DOI:** 10.1186/1471-2393-14-112

**Published:** 2014-03-24

**Authors:** Anne Debost-Legrand, Hélène Laurichesse-Delmas, Christine Francannet, Isabelle Perthus, Didier Lémery, Denis Gallot, Françoise Vendittelli

**Affiliations:** 1 Service de Génétique Médicale, Centre Hospitalier Universitaire de Clermont-Ferrand, Clermont-Ferrand, France; 2 Pôle de Gynécologie-Obstétrique et Reproduction Humaine, Centre Hospitalier Universitaire de Clermont-Ferrand, Clermont-Ferrand, France; 3 Clermont Université, Université d’Auvergne, EA 4681, PEPRADE (Périnatalité, grossesse, Environnement, PRAtiques médicales et DEveloppement), Clermont-Ferrand, France; 4 CEMC-Auvergne, Agence Régionale de Santé d’Auvergne, InVS, INSERM, Clermont-Ferrand, France; 5 AUDIPOG (Association des Utilisateurs de Dossiers informatisés en Pédiatrie, Obstétrique et Gynécologie), Faculté de Médecine RTH Laennec, Lyon, France; 6 R2D2-EA7281, Clermont Université, Université d’Auvergne, Clermont-Ferrand, France; 7 Place Lucie et Raymond Aubrac, Clermont-Ferrand, Cedex1 63003, France

**Keywords:** Congenital malformation, Medical practice assessment, Prenatal diagnosis, Pregnancy, Ultrasound screening

## Abstract

**Background:**

Congenital malformations occur in 3-4% of live births. Their prenatal detection is performed by ultrasound screening. Any announcement about a suspected malformation is a source of stress for the parents, and misdiagnosis during ultrasound screening can lead to expensive and sometimes iatrogenic medical interventions. In this study, we aim to determine the false-positive rate, first overall and then by anatomical system, of ultrasound screening for congenital malformations in the second and third trimesters of pregnancy.

**Methods:**

Our sample includes all children born between 1 January, 2006, and 31 December, 2009, in the French region of Auvergne, whose mother had a prenatal ultrasound diagnosis of a congenital malformation during the second or third trimester of pregnancy confirmed by a follow-up ultrasound examination by an expert consultant ultrasonographer. The study included 526 fetuses, divided in 3 groups: false positives, diagnostic misclassifications, and true positives. The rates of false positives and diagnostic misclassifications were calculated for the sample as a whole and then by anatomical system.

**Results:**

Overall, the false-positive rate was 8.8% and the rate of diagnostic misclassification 9.2%. The highest false-positive rates were found for renal and gastrointestinal tract malformations, and the highest diagnostic misclassification rates for cerebral and cardiac malformations. The diagnostic misclassification rate was significantly higher than the false-positive rate for cardiac malformations.

**Conclusion:**

The false-positive rate during prenatal ultrasound is not insignificant; these misdiagnoses cause psychological stress for the parents and overmedicalisation of the pregnancy and the child.

## Background

The French national health insurance fund reimburses three ultrasound examinations during pregnancy, at 11-13 weeks, 20-24 weeks, and 30-35 weeks, in accordance with national guidelines [1]. In 2010, there were 802 224 births in metropolitan France [2], from 796 066 deliveries and thus theoretically 2 388 198 ultrasound examinations. A national survey in 2010 showed a mean number of ultrasound examinations per pregnancy of 5 ± 2.5, up from 2003 when it was 4.5 ± 2.2 [3]. Congenital malformations, nonetheless, occur in only 3-4% of live births, as we see from the registries that monitor the incidence in various countries, either nationally or regionally. Consequently, an increasing number of ultrasounds are performed each year in France, although the number of pregnancies with anomalies is relatively small and has remained constant. For these reasons, it becomes important to look at the false-positive rate to assess our ultrasonographic practices.

Some publications based on these registry data have looked at the sensitivity of prenatal ultrasound screening in the general population [4-6], which rose from 41 to 60% during the 1990s and to 80% over the past decade [7,8]. In the general population, the false-positive rate, all systems combined, has been estimated at 0.5 to 33.0% [9-11]. A Spanish hospital-based study found an overall false-positive rate of 9.3%, regardless of gestational age [12]. This rate varies according to anatomical system [13]. Accordingly, an Italian study assessed it at 9-11% for surgically curable malformations of the digestive organs and chest [14]. Other studies have estimated it at 1.5 to 20.0% in comparing prenatally-identified malformations with post-mortem examination findings after terminations of pregnancy [15-24].

To our knowledge, no population-based study has analysed the false-positive rate by anatomical system and distinguished it (no malformation detected at birth despite prenatal diagnosis) from the rate of diagnostic misclassifications (not all of the malformations suspected prenatally were confirmed or error about the type of malformation affecting the organ at birth).

Because most pregnancies are at low risk, any announcement about a suspected malformation is a source of stress for the parents [25]. Moreover, any misdiagnosis can lead to expensive and sometimes iatrogenic medical interventions. Accordingly, reducing the number of false positives at anomaly scans as much as possible is essential.

The principal objective of our study was to determine the false-positive rate during the second and third trimesters of pregnancy in Auvergne, a region of France. Our secondary objectives were to assess the false-positive rate by anatomical system and the diagnostic misclassification rate.

## Methods

Auvergne is a rural region in central France, with a population estimated on 1 January 2009 at 1 343 964 inhabitants, that is, 2.2% of the population of metropolitan (European) France [26]. Auvergne has 10 maternity units (1 level III, 6 level II and 3 level I), coordinated by a perinatal network. It has only one multidisciplinary prenatal diagnostic centre (CPDP), which is located in the level III university hospital centre and works with both the perinatal network and the Auvergne study centre for congenital malformations (CEMC-Auvergne). The CPDP’s purpose is to promote access to all types of prenatal diagnosis, serve as a clinical and laboratory reference centre for patients and physicians, and provide opinions and advice concerning diagnosis, treatment and prognosis to the physicians and clinical pathologists who contact them when they suspect an anomaly in an embryo or foetus. All fetal ultrasound anomalies identified throughout the region are presented to the CPDP for their opinion during a weekly staff meeting, with remote participation and teleradiology, in accordance with national guidelines [27].

The CEMC-Auvergne registry is a regional, population-based registry monitoring malformations in some 13 500 annual births. Terminations of pregnancy are included regardless of term. Stillbirths are registered at a gestational age of 22 weeks or more. Live born infants with malformations are identified up to the age of 1 year through voluntary reporting by the region’s hospitals and searches of medical records of the maternity and paediatric units in the area. Regardless of the child’s vital status, confirmation of the malformation (or malformations) is obtained after birth, by any means (including pathology examination of foetus or child, in case of death), before it is included in the CEMC-Auvergne database.

A registry representative participates routinely at these weekly CPDP expert meetings and was thus able to collect the names of all the children with a malformation suspected before birth, who were thus “pre-included” in nominative paper records (”pre-inclusion form”). Permanent inclusion in the registry’s computerised database does not occur until after birth, following examinations intended to determine conclusively the presence or absence of the malformation or misclassification about the malformed organ.

The source population of this study comprised children whose mother had a prenatal diagnosis of a malformation after an ultrasound during the second or third trimester of pregnancy and who were born in Auvergne between 1 January 2006 and 31 December 2009.

Our sample included all those children whose mother underwent a follow-up (second-look) ultrasound by a CPDP ultrasonographer and whose file was considered at a multidisciplinary CPDP meeting. Live-born or stillborn children were included if they were delivered in a maternity unit in Auvergne after 22 completed weeks of gestation (≥ 22 weeks^+0 d^). Both the register and our study database include in utero fetal deaths resulting from terminations of pregnancy for fetal anomalies, in accordance with French law.

**Figure 1:**
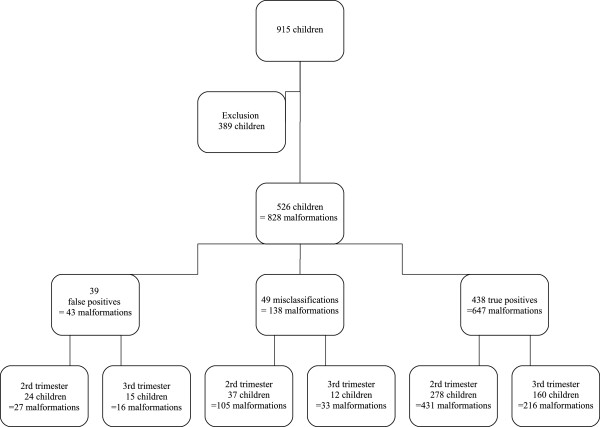
Selection of files for this study.

The exclusion criteria were the absence of pathology or other relevant examinations in cases of terminations of pregnancy or in utero fetal or early neonatal deaths, or the parents’ refusal to have a work-up that would confirm the prenatal diagnosis during the baby’s first year of life, or the impossibility of finding the results of work-ups that were in fact performed. Our final sample therefore included 526 children (Figure 1).

After birth (or terminations of pregnancy), the children were divided into three groups: the group of true positives, that is, those for whom malformations detected prenatally were confirmed during the postnatal examination; the group of false positives, that is, the children with a total discordance (no malformation identified at birth); and the group of misclassification errors (not all of the malformations suspected prenatally were confirmed by postnatal examination or the malformation of the suspected organ was confirmed at birth but the type of anomaly turned out to be erroneous). Ventriculomegaly was considered present if the width of the lateral ventricles, measured by ultrasound, ≥ 10 mm; it could be unilateral or bilateral. Pyelectasis was defined by an anteroposterior diameter of the renal pelvis > 5 mm between 20-29 weeks and >7 mm between 30-40 weeks, over several examinations. An abnormal quantity of amniotic fluid was defined as either oligoamnios [amniotic fluid index < 5th percentile] or polyhydramnios [amniotic fluid index > 95th percentile] [28]. Intrauterine growth restriction (IUGR) was defined as a birth weight <3th percentile for gestational age [29] according to the AUDIPOG (Association des Utilisateurs de Dossiers Informatisés en Pédiatrie, Obstétrique et Gynécologie) biometric curves [30].

The false-positive rate has as its numerator the number of children with a total discordance of one or more malformations identified in utero; its denominator is all 526 children in our study. The rate of misclassification errors was calculated as the ratio of the number of children with a partial discordance, again to all children in the study. To calculate the rates by anatomical system, only the files involving at least one malformation of the system considered were included, regardless of the number of systems with anomalies. Thus the files involving several systems were counted several times.

A Chi-2 test or Fisher’s exact test, as appropriate, was performed for the categorical variables. Analysis of variance (ANOVA) was used for the continuous variables. The level of statistical significance was set at 0.05. SAS software (version 9.1, SAS, Inc, Cary, NC, 2002-2003) was used to perform the analyses.

## Results

The final analysis included files for 526 children, prenatally diagnosed with 828 malformations. Malformations were diagnosed in 339 of these files during the second trimester and in 187 during the third (Figure 1).

Within this cohort, the mean maternal age was 29.4 (± 5.6) years. More than half (55.2%) had at least some university-level education. Smoking during the first trimester of pregnancy was noted for 28.5%. Maternal body mass index (BMI) ≥25 was reported for 11.6%. A degree of consanguinity with the spouse existed for 2.3% (Table 1). A history of familial congenital malformations was reported for 20.6% (Table 2). The mean number of previous pregnancies was 1.3 (+/- 1.5), and the rate of previous terminations 1.7%. The quantity of amniotic fluid was abnormal in 14.3% of the pregnancies (Table 2).

**Table 1:** Social and demographic data

	**Total cohort (n = 526) %**	**False positives (n = 39) %**	**Misclassification (n = 49) %**	**True positive (n = 438) %**	**p**^**1**^
**Maternal age**	(n = 526)	(n = 39)	(n = 49)	(n = 435)	
<25 years	18.1	19.2	22.5	17.5	0.62
25-34 years	62.3	65.9	59.2	62.3	
≥35 years	19.6	15.9	18.4	20.2	
**Educational level**	(n = 279)	(n = 22)	(n = 25)	(n = 232)	
Primary and middle school	24.6	20.8	20.0	25.4	0.81
High school	20.3	16.7	16.0	21.1	
Post-secondary	55.2	62.5	64.0	53.5	
**Smoking**	(n = 482)	(n = 40)	(n = 43)	(n = 399)	
	28.5	32.6	20.9	28.8	0.45
**BMI**^**2**^ **≥ 25**	(n = 526)	(n = 39)	(n = 49)	(n = 438)	
	11.6	8.5	0	13.2	0.02
**Consanguinity**	(n = 526)	(n = 39)	(n = 49)	(n = 438)	
	2.3	6.5	0	2.1	0.08

^1^Comparison of 3 groups: False positives, Misclassifications, True positives; ^2^BMI (Body Mass Index).

**Table 2:** Medical and obstetric data

	**Total cohort**	**False positives**	**Misclassification**	**True positive**	**P**^**6**^
	**(n = 526)**	**(n = 39)**	**(n = 49)**	**(n = 438)**	
	**% [m ± SD]**	**% [m ± SD]**	**% [m ± SD]**	**% [m ± SD]**	
**Number of pregnancies**^**1**^	(n = 526)	(n = 39)	(n = 49)	(n = 438)	
	[1.3 ± 1.5]	[1.1 ± 1.3]	[1.7 ± 1.8]	[1.3 ± 1.5]	0.38
**Miscarriages**	(n = 518)	(n = 39)	(n = 49)	(n = 430)	
	9.6	17.1	34.7	18.1	0.02
**Elective termination**	(n = 519)	(n = 39)	(n = 49)	(n = 431)	
	12.9	6.4	16.3	13.2	0.31
**TOPFA**^**2**^	(n = 517)	(n = 39)	(n = 49)	(n = 429)	
	1.7	4.3	2.0	1.4	0.16
**Nulliparous**	(n = 524)	(n = 39)	(n = 49)	(n = 436)	
	48.1	46.8	44.9	48.6	0.44
**Maternal diseases**^**3**^	(n = 520)	(n = 39)	(n = 49)	(n = 432)	
Diabetes	5.3	6.4	4.1	6.3	0.87
Epilepsy	0.9	0	0	1.2	0.37
Hypertension	0.8	2.1	0	0.7	0.35
**Familial malformations**	(n = 525)	(n = 39)	(n = 49)	(n = 437)	
Similar malformation	6.9	0	6.1	7.8	0.11
Different malformation	14.6	8.5	10.2	15.8	0.27
**Multiple pregnancy**	(n = 526)	(n = 39)	(n = 49)	(n = 438)	
	3.4	0	8.2	3.2	0.08
**ART**^**4**^	(n = 520)	(n = 38)	(n = 46)	(n = 436)	
	6.1	2.2	8.7	6.2	0.68
**PRD**^**5**^	(n = 508)	(n = 38)	(n = 47)	(n = 423)	0.64
	33.1	28.3	29.3	34.0	0.64
**Amniotic fluid anomaly**	(n = 516)	(n = 39)	(n = 49)	(n = 428)	
Oligoamnios	7.7	0	18.4	7.5	0.01
Hydramnios	6.6	2.1	8.2	6.8	

^1^Number of pregnancies, mean. ^2^ TOPFA (Termination Of Pregnancy for Fetal Anomaly**)**:In France, when a termination of pregnancy is envisaged for the reason that there is a strong probability that the child has a particularly severe condition recognized as incurable at the time of diagnosis, a multidisciplinary team from the authorised antenatal diagnostic center is responsible for examining the woman’s request and issuing an advisory opinion. If at the conclusion of this team’s assessment, two physicians consider that there is a strong probability that the child has such a condition, they complete a written attestation, one copy of which is provided to the woman. ^3^Maternal chronic disease (preceding the pregnancy) ^4^ART (Assisted Reproductive Technologies) ^5^PRD (Pregnancy related diseases including pregnancy related diabetes or hypertension, preeclampsia, etc.) ^6^Comparison of 3 groups: False positives, misclassifications, True positives.

Induced abortion occurred in 23.1% of the cases, and 71.7% of the children were live born (Table 3). The mean gestational age at the prenatal malformation diagnosis was 26.9 weeks (+/- 4.9). Each child had a mean of 2.1 (+/- 1.8) malformations in 1.5 (+/-1.1) anatomical systems (Table 4).

**Table 3:** Obstetric and neonatal outcome

	**Total cohort**	**False positives**	**Misclassification**	**True positives**	**p**^**1**^
	**(n = 526)**	**(n = 39)**	**(n = 49)**	**(n = 438)**	
	**% [m ± SD]**	**% [m ± SD]**	**% [m ± SD]**	**% [m ± SD]**	
**Year of birth**	(n = 526)	(n = 39)	(n = 49)	(n = 438)	
2006	22.5	17.0	26.6	22.5	0.49
2007	25.1	21.3	20.4	26.0	
2008	28.1	34.0	28.6	27.4	
2009	24.3	27.7	24.5	23.9	
**Female**	(n = 526) 45.9	(n = 39) 44.7	(n = 49) 48.9	(n = 438) 45.7	0.89
**Pregnancy outcome**^2^	(n = 526)	(n = 39)	(n = 49)	(n = 438)	
TOPFA< 21 weeks	2.3	0	4.2	2.3	0.39
TOPFA ≥ 22 weeks	20.8	0	31.3	21.9	0.0001
In utero fetal death	2.5	0	2.1	2.7	0.50
Neonatal death	2.7	2.1	2.1	2.7	0.94
**Term at birth (weeks)**	(n = 526)	(n = 39)	(n = 49)	(n = 438)	
	[35.5 ± 6.1]	[39.4 ± 1.9]	[34.2 ± 6.5]	[35.2 ± 6.2]	0.001
**Weight (g)**	(n = 526)	(n = 39)	(n = 49)	(n = 438)	
	[2588 ± 1134]	[3272 ± 525]	[2265 ± 1211]	[2551 ± 1147]	0.001
**IUGR (3**^**rd **^**Percentile)**	(n = 482)	(n = 39)	(n = 44)	(n = 399)	
	4.9	2.1	9.1	4.8	0.03
**Height (cm)**	(n = 526)	(n = 39)	(n = 49)	(n = 438)	
	[45.1 ± 7.7]	[49.3 ± 2.1]	[42.2 ±8.9]	[44.9 ±7.8]	0.001
**Type of malformation**	(n = 526)	(n = 39)	(n = 49)	(n = 438)	
Single	76.0	100	53.1	76.3	0.40
Multiple	8.2	0	22.5	7.3	
Syndrome with normal karyotype	4.3	0	8.2	4.3	
Chromosomal anomalies	11.4	0	16.3	12.1	

^1^Comparison of 3 groups: False positives, Misclassifications, True positives.

^2^TOPFA (Termination Of Pregnancy for Fetal Anomaly).

**Table 4:** Characteristics of malformations

	**Total cohort**	**False positive**	**Misclassification**	**True positive**	**p**^**1**^
	**(n = 526)**	**(n = 39)**	**(n = 49)**	**(n = 438)**	
	**% [m ± SD]**	**% [m ± SD]**	**% [m ± SD]**	**% [m ± SD]**	
Term at prenatal diagnosis or malformation (weeks):	(n = 526) [26.9 ± 4.9]	(n = 39) [27.2 ± 5.2]	(n = 49) [26.1 ± 4.8]	(n = 438) [26.9 ± 4.9]	0.45
20-25	50.9	46.8	58.6	50.0	0.92
26-29	13.1	10.6	20.7	13.5	
30-35	32.4	38.3	20.7	33.1	
>35	3.6	4.3	0	3.4	
Number of malformations	(n = 526)	(n = 39)	(n = 49)	(n = 438)	
1	51.7	93.6	6.1	52.3	0.0001
2	21.9	4.3	32.7	22.6	
3	13.7	2.1	22.5	13.9	
≥ 4	12.7	0	38.8	11.2	
Number of anatomical systems affected	(n = 526)	(n = 39)	(n = 49)	(n = 438)	
1	71.9	100	48.9	71.9	0.0001
2	13.5	0	20.4	13.9	
3	6.7	0	6.1	7.3	
≥ 4	7.9	0	24.5	6.9	

^1^Comparison of 3 groups: False positives, Misclassifications, True positives.

Tables 1 to 4 summarise the social, demographic, medical, obstetric and ultrasonographic data of mothers and children. There was no difference between the three groups for mothers’ characteristics, except for maternal obesity, which was more frequent in the true-positive group (p = 0.02) (Table 1). Previous spontaneous abortions were more often reported in the misclassification group (p = 0.02), as was oligohydramnios (p = 0.01) (Table 2). Terminations of pregnancy after 22 weeks were reported more often in the misclassification group (p = 0.0001). Gestational age at birth was higher in the group of false positives (p = 0.001). Weight and height at birth were also higher in the false-positive group, compared with the two other groups (p = 0.001). Finally, intrauterine growth restriction (IUGR) was more frequent in the misclassification group (p = 0.03) (Table 3). There were more isolated malformations in the false-positive group and more multiple malformations (≥4) in the diagnostic misclassification group (p = 0.0001). Moreover, in general, only one organ was affected in the false-positive group, and several anatomical systems were involved in the misclassification group (p = 0.0001) (Table 4).

Among the 526 children, the false-positive rate was 8.8% (95% CI: 6.5-11.5) after the specialized follow-up ultrasound, regardless of the term at ultrasound. The false-positive rate was 7.9% (95% CI: 5.3-11.3) when the malformation was screened during the second trimester and the specialized follow-up ultrasound performed between 20 and 30 weeks, and 10.4% (95% CI: 6.5-15.6) when the malformation was screened during the third trimester and the specialized follow-up ultrasound performed after 30 weeks.

The overall diagnostic misclassification rate was 9.2% (95% CI: 6.9-11.9); when the malformation was screened during the second trimester and the specialized follow-up ultrasound performed between 20-30 weeks, it was 10.8% (95% CI: 7.7-14.6), and when screening took place during the third trimester and the specialized follow-up ultrasound after 30 weeks, 6.3% (95% CI: 3.3-10.7).

Table 5 presents the rates of false positives and diagnostic misclassifications according to the affected anatomical system. Only the cardiovascular system showed a significant difference between the false-positive group and the diagnostic misclassification group, with a higher proportion of cardiac malformations in the latter (p = 0.01).

**Table 5:** Rates of false positives and diagnostic misclassifications by anatomical system (number of files)

**Malformations identified in utero**	**False positives**	**Misclassification**	**p**^**4**^
	**% (95% CI)**	**% (95% CI)**	
Bone malformations (n = 56)	3.6 (0.04-12.3)	12.5 (5.2-24.1)	0.10
Cardiac malformations^1^ (n = 86)	3.4 (0.07-9.5)	14.6 (8.0-23.7)	0.01
Cerebral malformations^2^ (n = 98)	7.1 (4.2-16.4)	15.3 (8.7-23.5)	0.22
Malformations of the respiratory system and intrathoracic organs (n = 16)	6.3 (1.5-36.4)	0	0.16
Craniofacial malformations (n = 45)	4.4 (5.4-15.1)	15.6 (6.5-29.5)	0.09
Malformations of the eyes (n = 4)	0	25 (0-67.4)	0.31
Malformations anterior abdominal wall (n = 14)	7.1 (0-20.6)	21.4 (4.6-50.8)	0.32
Malformations of genital organs (n = 35)	5.7 (2.4-28.1)	7.9 (1.7-21.4)	0.48
Renal malformations ^3^(n = 169)	11.8 (7.4-17.7)	13.6 (8.8-19.7)	0.65
Malformations of the gastrointestinal tract (n = 41)	12.2 (4.1-26.2)	9.8 (2.7-23.1)	0.73

^1^The specific cardiac anomaly most often misdiagnosed was ventricular septal defect (n = 5).

^2^The specific cerebral anomalies most often misdiagnosed were anomaly of the corpus callosum (n = 5) and ventriculomegaly (n = 5).

^3^The specific renal anomaly most overdiagnosed in the false-positive group was renal pyelectasis (n = 15).

^4^Comparison between 2 groups: False positives, Misclassification.

## Discussion

This study reports false-positive and diagnostic misclassification rates during ultrasound screening in the general population over a 4-year period. The false-positive rate in this study (8.8%) is similar to that in recent data from the literature, with rates between 9 and 12% [9,12,22,31]. The oldest studies reported higher rates (up to 33%) [10]. This rate therefore varies according to the study period: as prenatal diagnosis has improved with time, the false-positive rate has become lower in the most recent studies and is now consistent with our rate.

The rate of diagnostic misclassifications in our study (9.2%) seems higher than those found in the literature, which are around 3% [19-21]. This discordance may be explained by our study’s coverage of two trimesters of pregnancy; the literature appears to consider only the second trimester (12-28 weeks) [15-24]. Moreover, the series in the literature concern only cases of induced abortions for severe malformations, which are most often well identified. We note that the cases with terminations in the misclassification group in our study had all multiple anomalies and that the incorrectly diagnosed malformations would not have influenced medical decision. The objective of this paper was to describe the global rate of false positives, that is, all diagnoses of malformations that turned out not to be present. It is important to note that the false positive diagnoses in the misclassification group may wrongly evoke suspected syndromes. Thus while they may not influence the medical outcome of the pregnancy, they may nonetheless induce specific unnecessary complementary examinations, be a source of stress and have costs for society.

These rates are based on data from one regional prenatal diagnosis centre, where all pregnancies with suspected foetal malformations are assessed, contrary to published studies that compare several hospitals of different levels [9,22].

Somewhat surprisingly, the proportion of women with a BMI ≥ 25 was higher in the true-positive group than in the other two groups. This result is discordant with that of the FASTER trial, which showed an inverse relation between maternal BMI and the false-negative rate [32]. This overrepresentation of overweight women in the group of true positives might be related to their overrepresentation in the group with malformed children, because of the teratogenic effect of some maternal diseases found more frequently in this population (especially diabetes). It is impossible to study the predictive factors for false positive or misclassified diagnoses because our database includes only the children with a prenatal diagnosis of malformation; the medical, social and demographic characteristics of the pregnancies studied might thus be different from those of pregnancies with no anomalies.

Most of the malformations in the false-positive group were isolated, as previously reported in the literature [12]. They thus differ from the diagnostic misclassification category, where there are more often multiple malformations involving several anatomical systems. This characteristic is also present in studies of correlations between prenatal data and autopsy diagnoses, where most of the fetuses have multiple malformations, which can induce some difficulties in interpreting the ultrasonographic images [10,22]. It has already been reported that the value of ultrasound is limited in situations with multiple malformations [23,33]. Complementary imaging by nuclear magnetic resonance (NMR) appears more effective in some cases [33]. As a consequence, terminations of pregnancy were more frequent in the misclassification group as the overall malformations were more severe than in the other two groups. No termination of pregnancy was performed for any of the malformations in the false-positive group, for none of them suggested that “there was a strong probability that the child to be born would have a severe condition recognized as incurable at diagnosis,” the criterion for termination under French law.

Oligohydramnios was also present more often in the misclassification group; as already reported, oligohydramnios may lead to inconclusive ultrasonographic diagnoses [34]. Finally, IUGR affected more children in the misclassification group; it has previously been demonstrated that risk of IUGR is frequently associated with a high number of malformations [35].

The study of the false-positive rate by anatomical system shows that these were most often gastrointestinal tract and kidney malformations. These results are equivalent to those in the Eurofetus study, where anomalies of the urinary tract and gastrointestinal system respectively accounted for 16.5% and 15.2% of the false-positive diagnoses [9] and in the Spanish hospital-based study, where urinary tract anomalies had the highest absolute number of false positives and those of the gastrointestinal system had one of the highest false-positive rates (11.6%) [12].

It has now been demonstrated that the course of pyelectasis depends on its size [36]. Dilatation measured at 15 mm prenatally during the third trimester appears to be the threshold value for suspecting the presence of renal dysplasia or an underlying uropathy at birth. For dilatation of 10 mm, spontaneous resolution is observed in nearly 35% of cases, and for dilatation less than 10 mm, resorption occurs in nearly 50% to 80% of cases [37,38]. In our study of the 15 prenatal cases of isolated pyelectasis not observed after birth, 12 had a renal pelvis smaller than 10 mm. However, pyelectasis can disappear because of newborn dehydration at birth and be detected at a later check-up. Infants in our study were followed up to one year of age and thus received the maximum duration of postnatal renal monitoring.

Data about diagnostic misclassifications are sparse; they are grouped most often in the literature with the false positives [19,21,23]. Studies of the correlation between prenatal ultrasound scans and pathology examinations show that diagnostic misclassifications principally concern heart disease [18,19,21], with the most frequent diagnostic error that of a ventricular septal defect, which often marks valvular disease [19,39]. Similarly, it has already been shown that postnatal pathology examinations fail to confirm a substantial portion of cerebral malformations [23]. Moreover, moderate ventriculomegaly (with a size between 10 and 15 mm) may be a type of functional anomaly that sometimes regresses during pregnancy [40,41]. The ventricle size of the cases of isolated prenatal ventriculomegaly not confirmed after birth in our study ranged from 10 to 13 mm. It has been demonstrated that early cerebral autolysis can also mask the presence of an anomaly during the postnatal period and prevent diagnostic confirmation of the prenatal imaging [23].

Corpus callosum agenesis is the most frequent commissural malformation. The false-positive rate of ultrasound screening for this malformation varies from 0 to 22%. It may be over-diagnosed because of its association with ventriculomegaly, which may hinder adequate visualization of the cerebral structures [42]. Three-dimensional sonography makes it possible to acquire the foetal head volume from the axial view and then, by reconstructing the image with the multiplanar technique, to obtain a suitable view of the corpus callosum without the technical difficulties of 2D-sonography. Second-step complementary NMR remains, however, a clinically valuable adjunct to ultrasound and provides additional information when the ultrasound diagnosis is uncertain [43,44].

One limitation of our study is that the CEMC database registers only children in whom malformations have been confirmed after birth or induced abortion, so that it is not possible to analyse the predictive values of the ultrasound screening. Another limitation may be the absence in our database of reliable data about the mode of dating pregnancies: this information is missing for many women. Nonetheless, in France the national health insurance fund reimburses three ultrasound examinations during pregnancy; the first is performed between 11 and 14 weeks to determine term and measure nuchal thickness as part of trisomy 21 screening. Accordingly, the pregnancy date is most often determined by fetal ultrasound. The national survey perinatal found that only 0.1% of women had no ultrasound during pregnancy in 2003, and 0.2% in 2010 [45]. It is therefore unlikely that our results are biased (as ultrasonographic term is correlated with the beginning of pregnancy) by the absence of data about early fetal ultrasound.

## Conclusion

The prenatal ultrasound false-positive rate in the region of Auvergne during the second and third trimesters was 8.8%. This rate varied according to the anatomical system. These erroneous diagnoses have psychological repercussions for families and also engender costs for society, due to the futile medical examinations. Continuation of this work should seek to identify the factors that promote false-positive prenatal ultrasound findings.

### Ethical approval

IRB 5921 (CECIC, ethics committee) for Rhône-Alpes-Auvergne (Grenoble) approved this study on June 19, 2012. The database has been reported to the French Data Protection Authority (CNIL: Commission Nationale de l’Informatique et des Libertés) as CNIL n° 1387396.

## Abbreviations

CPDP: Multidisciplinary prenatal diagnostic centre; CEMC-Auvergne: Auvergne study centre for congenital malformations; CNIL: Commission Nationale de l’Informatique et des Libertés; BMI: Body mass index; IUGR: Intrauterine growth restriction; NMR: Nuclear magnetic resonance; TOPFA: Termination of pregnancy for fetal anomaly; PRD: Pregnancy-related diseases; ART: Assisted reproduction technologies.

## Competing interests

The author declares that they have no competing interests.

## Authors’ contributions

ADL and FV conceived and designed the study. ADL performed statistical analyses. FV validated the results. ADL drafted the manuscript, FV supervised manuscript modification. HDL, CF, IP, DL and DG carried out clinical assessments. All authors contributed to the interpretation and writing of the paper and approved the final version.

## Pre-publication history

The pre-publication history for this paper can be accessed here:

http://www.biomedcentral.com/1471-2393/14/112/prepub
